# A novel wrinkled-leaf sesame mutant as a potential edible leafy vegetable rich in nutrients

**DOI:** 10.1038/s41598-022-23263-0

**Published:** 2022-11-02

**Authors:** Hongyan Liu, Fang Zhou, Ting Zhou, Yuanxiao Yang, Yingzhong Zhao

**Affiliations:** Key Laboratory of Biology and Genetic Improvement of Oil Crops, Ministry of Agriculture, Oil Crops Research Institute of Chinese Academy of Agricultural Sciences, Wuhan, 430062 Hubei China

**Keywords:** Plant sciences, Plant physiology

## Abstract

Sesame (*Sesamum indicum* L.) is an ancient and globally important oil crop in the tropic and subtropic areas. Apart from being a good source of high-quality oil, sesame also represents a new source of edible leafy vegetables. However, data regarding the nutritional composition of the sesame leaves, especially their phytonutrient composition, are scarce. Previously we have developed a sesame mutant JQA with curly, wide, and thick leaves that are potentially used as a vegetable. The objective of this work was to gauge the nutrient contents in leaves of the JQA mutant by colorimetry methods. The sesame mutant JQA and its wild-type counterpart JQB were grown in the field, and leaf samples were collected at the flowering stage. Results showed that the sesame wrinkled leaves of JQA are a rich source of crude oil (5.33–6.38%), crude protein (3.14%), amino acids (> 18.6 mg/g), crude fiber (> 0.36%), cellulose or hemicellulose (> 21.4 mg/g), sugars (> 12.5 mg/g), vitamins, and flavones (> 63.2 mg/g). The wrinkled sesame leaves were high in unsaturated acid (32.0 mg/g), calcium (18.5 mg/g), potassium (16.1 mg/g), as well as vitamin B_6_ (24.5 mg/g), B_2_ (14.4 mg/g), C (1.7 mg/g) and D (1.3 mg/g) compared to other common green leafy vegetables. The fresh leaves had a mean total flavone content of 65.7 mg/g and can be consumed as fresh vegetables or preserved in a dry state. Collectively, the nutritional composition of the wrinkled leaf mutant JQA was ideal and thus had high RDIs (recommended daily intakes), suggesting that the wrinkled leaves are a rich source of nutrients and therefore suitable to be consumed as a new edible green vegetable.

## Introduction

Sesame (*Sesamum indicum* L.) is an ancient and globally important crop cultivated mainly in developing countries such as Tanzania, Myanmar, India, and China in tropical and subtropical areas^1,2^, with a total sesame seed production of over 6.0 million tons in recent years (UN Food and Agriculture Organization Statistical Databases, 2020). In China, although sesame can be grown across the country due to its resilience to the local growth climate and environments, it is cultivated predominantly in the central part (namely Henan, Hubei, Anhui, and Jiangxi provinces) with a total output of 50,000 tons per year^3^. Sesame seeds are highly nutritious (oil 40–60%, protein 15–25%), and the oil is rich in natural antioxidants such as sesamin and sesamol^4,5^, which have specific antihypertensive effects and antioxidative activity^6,7^. Sesame leaves are a byproduct of sesame oil production and can be consumed as a cold dish, in a pancake, or with noodles^8^. In addition to being a fresh food, the leaves can be dried or canned^9^. Generally, sesame leaves are flat and have a bitter taste, but also have extensive medicinal value. The leaves function in nourishing the kidney and liver, lubricating the intestines, and alleviating constipation, and can also be used to treat hair loss, dyschezia, and acute and chronic pharyngitis^10^. In recent years, sesame leaf has gained popularity as a unique, delicious, and economical vegetable because of its refreshing taste and smooth texture, versatility, and high medicinal value^11^.

Traditionally, sesame leaves have long been recognized as a specialty vegetable in some areas of China, especially Henan and parts of Hubei province. Typically, a sesame plant produces 50–80 leaves; the leaves on the middle and lower part (one-third of the plant height), which can be harvested at the end of the flowering stage, are suitable for consumption because of their large area^12^. Approximately 120,000 plants can be cultivated per hectare in summer, and each plant can yield about 100 g of fresh leaves or 20 g of dried leaves (the petiole is excluded). Thus, the fresh leaf yield can be as high as 1.2 tons/ha and is an important target trait for breeding^12^. We previously identified a sesame mutant with wrinkled, big, and tender leaves that have a better taste than the wild type. There were no obvious differences in other agronomical characters between the mutant and wild type^13^. The wrinkled-leaf mutant can be used as a vegetable because of its high biological yield and good flavor. This attractive property makes it highly valued for application in the future.

Generally, leafy green vegetables can provide substantial amounts of vitamins, minerals, proteins, and fibers, as well as antioxidant phytonutrients that play a pivotal role in the prevention or mitigation of obesity, cardiovascular disease, and other diseases including cancer^14^. The nutrient value of vegetables is often determined by Recommended Daily Intakes (RDIs). Kale is a vegetable with high RDIs (35 cal, 6 g of carbohydrates, 3 g of protein, and high levels of antioxidants)^15^.

Sesame leaf is another important vegetable resource especially for the poor populations in low-income countries, providing nutrients that are indispensable to the human body^10,11^. However, studies on the nutritional value of sesame leaves were very limited so far, although there were some investigations on the extraction of total flavonoids^17^, measurement of mineral elements^18^, and the abundance of active polysaccharides^19^. In this study, the contents of crude oil and fatty acid compositions, soluble protein and amino acids, macro- and micro-mineral elements, as well as vitamins and reducing sugar in the leaves of the wrinkled-leaf mutant JQA were analyzed. We expect to provide a reference of nutrition value for the further development and utilization of sesame leaf resources.

## Materials and methods

### Plant materials

The sesame plant materials used in this study included JQA (a wrinkled-leaf line) and a wild-type line with normal leaf (JQB) (Supplementary Fig. 1). The details of developing JQA and JQB have been reported previously^13^. Briefly, a wrinkled-leaf plant was identified in an F_2_ segregation population, which was then sib-mated 5 to 7 times to obtain highly homozygous near-isogenic lines JQA and JQB. All plant materials were grown from 2011 to 2018 in Wuhan (in the experimental station of the Oil Crops Research Institute, Chinese Academy of Agricultural Sciences) in summer but in Sanya during winter, to speed up the breeding progress. All leaf samples were collected in Wuhan in 2018 for analysis with three biological replications. Plants were arranged in rows 2.4 m apart and with 0.1-m plant spacing. Field management followed normal agricultural practices during the growing season. All the experimental research and field studies on plants comply with relevant institutional, national, and international guidelines and legislation.

### Determination of total nitrogen

The content of total nitrogen in the plants was determined using the micro-Kjeldahl method with hydrogen peroxide digestion^20^. The dried plant leaves were boiled with concentrated sulfuric acid and hydrogen peroxide to convert the organic nitrogen into ammonium salt. The ammonium salts formed into ammonia after alkalization, and then the ammonia was absorbed into the boric acid solution by distillation. The total nitrogen content of the plant leaves (not including all nitrate nitrogen) was determined by standard acid titration using methyl red-bromocresol green as the indicator.

### Determination of macro- and trace-element contents

The contents of macro elements and trace elements were determined by inductively coupled plasma-atomic emission spectrometry (ICP-AES) with the nitric acid-hydrogen peroxide digestion method^21^. About 0.2 g of dried leaf powder was placed into a closed vessel microwave digestion system (MLS-ETHOS plus), following which 5 mL of nitric acid and 2 mL of hydrogen peroxide were added, and the solution was digested for 20 min in a microwave using the following conditions: 6 min for 250 W, 6 min for 400 W, 8 min for 550 W, and vent for 8 min. A blank digest was performed in the same way as technical control. After the digest solution became clear, it was diluted to 50 mL with ddH_2_O water and then filtered. The content of each element was ultimately determined on an X2 ICP-MS machine.

### Determination of soluble protein and crude oil contents

The content of soluble protein was determined by the colorimetry Coomassie brilliant blue G250 staining method. Approximately 0.5 g of fresh sesame leaf sample was grinded into homogenate, dissolved in 6 mL distilled water, incubated at room temperature (20–25 °C) for 1 h, and then centrifuge at 4000 r/min for 20 min. The supernatant was transferred to a flask and brought to a final volume of 10 mL with distilled water. Finally, 1 mL of supernatant was mixed with 5 mL of G250 solution (100 mg/L), and the absorbance was determined at 595 nm on a UV–visible spectrophotometer (TU-1810D, Beijing, China). The concentrations were inferred from a standard bovine serum albumin (BSA) curve. The crude oil content was determined by extracting a known weight of the sample (5 g) with ether (80 °C) for 12 h using the Soxhlet extractor^22^.

### Determination of soluble sugar, reducing sugar, and total sugar contents

The content of soluble total sugar was determined by the sulfuric acid–anthrone method^22^; in brief, 0.10 g of crushed leaf sample was dissolved in 10 mL distilled water, extracted in boiling water for 30 min (twice), then filtered and adjusted to a final volume of 25 mL by adding pure water. Next, 0.5 mL of this sample solution was mixed with 1.5 mL of distilled water, 0.5 mL of ethyl anthrone acetate reagent, and 5 mL of concentrated sulfuric acid. The mixture was kept in a boiling water bath for 1 min and then naturally cooled down to room temperature before measurement. The absorbance was detected at the wavelength of 630 nm, from which the amount of soluble sugar (μg) was calculated by comparing it with the standard linear equation based on sugar. Likewise, the reducing sugar was determined by the 3,5-dinitrosalicylic acid colorimetric method^22^, and the total sugar content was determined by the hydrochloric acid hydrolyzed-anthrone colorimetric method^23^.

### Determination of fatty acid content

The content of fatty acids was determined by gas-phase mass spectrometry^24,25^. About 200 mL of extracted crude oil was saponified with 3.0 mL of 0.5 mol/L KOH in MeOH by heating and stirring for 5 min at 100 °C in a volumetric flask. After saponification, the oil was esterified by adding 2.0 mL 14% boron trifluoride, and boiled for 5 min to form fatty acid methyl esters, which were then extracted by first adding 2.0 mL of *n*-hexane and later 5 mL saturated NaCl solution; the solution was mixed carefully and thoroughly and allowed settling for 20 min. After separation, the liquid supernatant (*n*-hexane phase) was transferred to a vial for fatty acid methyl esters analysis using an Agilent Ag1100 liquid chromatography. The model of the gas-phase capillary column was Agilent SP-2560 (100 m × 0.25 mm × 0.2 μm film thickness). The initial temperature of the column was set at 70 °C, then was increased to 140 °C for 1 min at the rate of 50 °C/min; then it was further raised to 180 °C for 1 min at the rate of 4 °C/min, and finally to 225 °C for 30 min at the rate of 3 °C/min. The temperature of the gasification chamber was 250 °C. N_2_ was used as the carrier gas in the column at a flow velocity of 1 mL/min and a split ratio of 45:1. About 1 μL of the prepared sample was loaded, and the resultant peaks were identified by comparing retention times with authentic fatty acid methyl esters. Quantification was based on the area under each fatty acid peak as compared to the total area of all fatty acid peaks.

### Determination of cellulose, hemicellulose content, lignin content, and crude fiber

The content of cellulose was determined by the anthracene sulfate colorimetry method^26^; briefly, 100 mg of the air-dried sample was put into a glass bottle containing 100 mL of cold H_2_SO_4_ solution (60% v/v), digested for 30 min under cold conditions, filtered with a glass crucible hopper, and from which 5.0 mL solution was transferred to a new bottle and diluted to 50 mL with ddH_2_O. Next, 2.0 mL of the supernatant was mixed with 0.5 mL of anthrone reagent (2%, w/v), as well as 5.0 mL of concentrated H_2_SO_4_ solution in a new tube. The mixture was shaken gently to promote the hydrolysis of ethyl acetate. When the anthrone flocculent appears, the tube was shaken violently to promote the dissolution of anthrone, then immediately heat for 10 min in boiling water and then cool down before measurement. The concentration of cellulose was determined by comparing the absorbent value of the sample solution at 620 nm on a spectrophotometer with those of a standard curve. Similarly, the hemicellulose content was determined by the 2% hydrochloric acid hydrolysis method combined with the 3, 5-dinitrosalicylic acid (DNS) reducing sugar content determination method^26^; the lignin was determined by the oxidation–reduction titration method^26^; the crude fiber was determined using the improved acid washing method^27^.

### Determination of hydrolytic amino acid components

The content of hydrolyzed amino acids was determined by liquid chromatography^28^. The extracted sample was tested on an Agilent Ag1100 liquid chromatography (American Agilent Company). The chromatographic column was ODS HYPERSIL (250 m × 4.6 mm, 5 μm). The column temperature was set at 40 °C, with a flow rate of 1.0 mL/min. The Double channel ultraviolet UV detector (Pro, Hypro) was set at a wavelength of 338 nm and 262 nm, respectively.

### Determination of total flavonoids

The content of total flavonoids was determined by spectrophotometry^29^. Using absorbance as the ordinate and the concentration of the rutin control solution as the horizontal, a standard curve was drawn. Precisely, fresh leaves weighing 0.3 g were ground to a homogenate and used for sample extraction. The concentration of rutin in the sample solution was measured under a wavelength of 510 nm on a spectrophotometer. The content of total flavone was then calculated.

### Determination of vitamin C, A, D, B_1_, B_2_, and B_6_ content

The content of vitamin C was determined by the molybdenum blue colorimetry method^30^. Briefly, 0.5 g of leaf sample was grinded and dissolved in 10 mL of oxalic acid-EDTA solution, then filtered, and 5 mL of the filtrate was diluted into 50 mL with oxalic acid–EDTA solution. Next, 10 mL of such solution was transferred into a new flask, mixed with 1 mL of the metaphosphoric acid solution, and 2 mL of sulfuric acid (5%, v/v). The mixture was shaken, then 4 mL of ammonium molybdate and 33 mL of distilled water were added to get a final volume of 50 mL. After inoculation at 30 °C for 20 min, the absorbance value was measured at 760 nm and the concentration of vitamin C was calculated by comparing it with a standard curve. Similarly, vitamins A and D were determined by high-performance liquid chromatography-tandem mass spectrometry^31^, and vitamins B_1_, B_2_, and B_6_ were determined by high-performance liquid chromatography^32^.

### Biological traits investigation

We measured the agronomic traits of the wild type and mutant. During the full blossom stage, each of the 10 plants from the JQA and JQB lines was selected for the investigation of leaf morphological traits. To determine the mean length and width of a leaf, the largest one on each plant was picked and their two linear dimensions were measured using a Vernier caliper with an accuracy of 0.1 mm (Canon Instruments, Japan). The yield of fresh leaves was estimated by weighting all leaves collected from three independent plots (each with 66.7 m^2^) when the first capsule is mature.

### Statistical analysis

All samples were analytically tested with three biological replications. The values of different parameters were expressed as the mean ± standard deviation. Comparison of means was performed by one-way analysis of variance (ANOVA) followed by Wilcoxon’s multiple comparison tests. Statistical significance was set at the 5% and 1% levels of probability using IBM SPSS Statistics V19 software (IBM, Armonk, New York, USA).

### Ethical approval

This article does not contain any studies with human participants or animals performed by any of the authors.

## Results and discussion

### Crude oil content and fatty acid compositions

The crude oil content in the seeds of sesame produced in China is about 52–62%^3^; however, the content in the leaves has not yet been determined. Our results showed that the crude oil in the leaves of the wrinkled-leaf mutant JQA and its wild-type JQB was 5.3% and 6.4% on average, respectively (Table 1). This result suggests that sesame leaf is a good source of oil that can be exploited in the future. The content in the mutant JQA was significantly lower (by 1.1%) than that in the wild type (*P* < 0.05), but as a result, the wrinkled leaves tasted better when cooked due to their lower crude oil content (Supplementary Fig. 2).

**Table 1:** Crude oil content and fatty acid compositions in sesame leaves.

Parameters^a^	Wild type JQB	Wrinkled-leaf mutant JQA^b^
Crude oil (%)	6.38 ± 0.32	5.33 ± 0.22*
**Fatty acid composition (mg/g)**		
C11:0	4.863 ± 0.251	4.863 ± 0.223
C12:0	0.044 ± 0.007	0.032 ± 0.006
C14:0	0.544 ± 0.024	0.408 ± 0.006*
C15:0	0.212 ± 0.010	0.146 ± 0.007*
C16:0	20.011 ± 0.986	15.655 ± 0.517**
C16:1	0.318 ± 0.013	0.288 ± 0.025
C17:0	0.098 ± 0.022	0.089 ± 0.013
C18:0	1.567 ± 0.113	1.469 ± 0.054
C18:1	1.162 ± 0.103	0.942 ± 0.031
C18:2	4.350 ± 0.034	3.946 ± 0.097*
C18:3	33.669 ± 1.076	26.836 ± 1.139**
C20:0	1.203 ± 0.127	1.035 ± 0.115
C22:0	1.094 ± 0.086	0.787 ± 0.058*
C24:0	0.282 ± 0.032	0.308 ± 0.010
Total	69.417 ± 2.522	56.803 ± 1.808*
Subtotal of saturated fatty acid	29.918 ± 1.477	24.791 ± 0.518*
Subtotal of unsaturated fatty acid	39.499 ± 1.212	32.012 ± 1.291*

^a^The details of fatty acid compositions can be found in Fig. 1.

^b^Values are means ± standard deviation for *n* = 3. Data in the same row followed by different letters are significantly different (*p* < 0.05).

Data in the same row followed by * and ** are significantly (*p* < 0.05) and very significant (*p* < 0.01) different, respectively.

**Figure 1:**
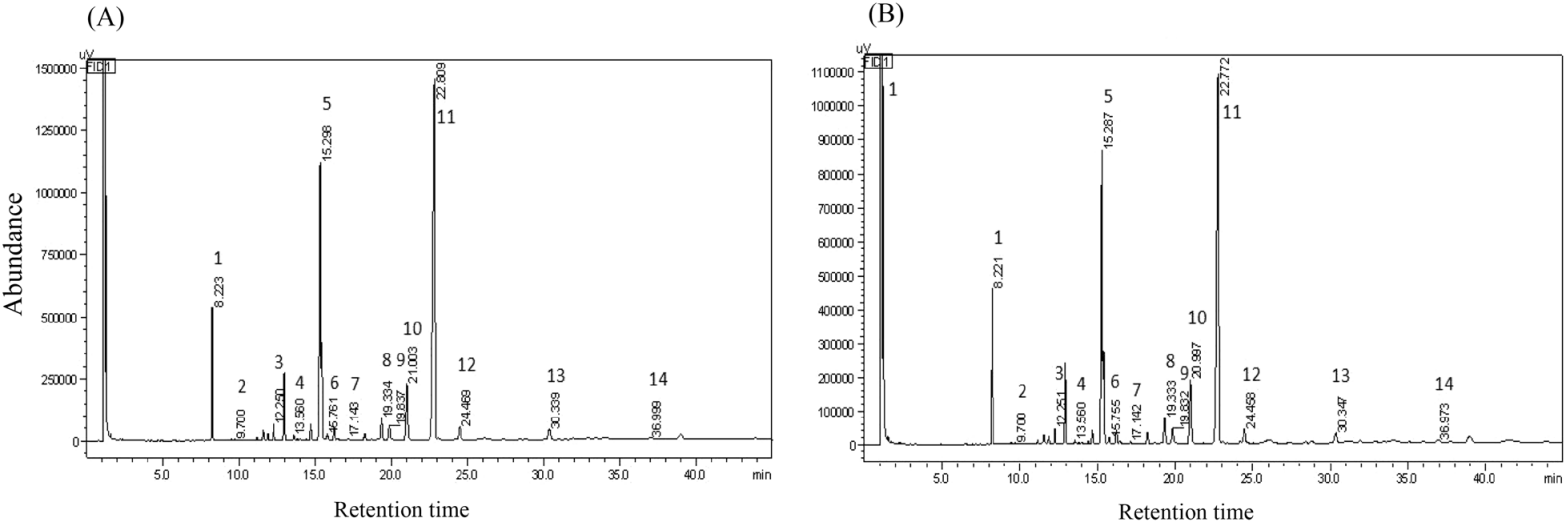
Representative gas chromatograms of the fatty acids in sesame leaves on a DB-5ms column. (**A**) Leaf sample prepared from JQA. (**B**) Leaf sample prepared from JQB. The optical density absorbance is plotted against retention time, and 14 peaks (numbering from 1 to 14) representing different fatty acid compositions were identified by comparing the retention time with that of standard fatty acid compounds. The absolute content (mg/g) for each fatty acid can be inferred from a linear regression established by plotting the injected amount of each fatty acid standard against the obtained peak area of the derivatives. Peak identity was as the following. 1: Undecylic acid (C11:0); 2: Lauric acid (C12:0); 3: Myristic acid (C14:0); 4: Pentadecane acid (C15:0); 5: Palmitic acid (C16:0); 6: Palmitate acid (C16:1); 7: Heptadecanoic acid (C17:0); 8: Stearic acid (C18:0); 9: Oleic acid (C18:1); 10: Linoleic acid (C18:2); 11: Linolenic acid (C18:3); 12: Arachidic acid (C20:0); 13: Elm acid (C22:0); 14: Paraffinic acid (C24:0).

We then determined the fatty acid compositions of the crude oil. Figure 1 shows the typical gas-phase mass spectrometry results for fatty acids in the leaves of JQA and JQB. Fourteen fatty acids in the leaves were identified and then quantified, and four of these (i.e., linolenic acid C18:3, palmitic acid C16:0, undecylic acid C11:0, and linoleic acid C18:2) were the major fatty acids. These four fatty acids added up to 51.3 mg/g (90.3% of the total of 14 fatty acids) and 62.9 mg/g (90.6% of the fourteen fatty acids) in the dry leaves of the mutant and wild type, respectively (Table 1, Supplementary Fig. 3). It has been reported that some of these fatty acids are beneficial to human health^33^. The highest content was found to be C18:3; in the dry leaves of the mutant and the wild type they were as high as 26.8 mg/g (accounting for 47.3% of the sum of fourteen fatty acids) and 33.7 mg/g (48.5% of the sum of fourteen fatty acids), respectively (Supplementary Fig. 3), which were much abundant than other oil crops such as rapeseed (1.0–3.5%)^34^. As is well documented, C18:3 is one of the essential fatty acids processing many physiological functions, such as reducing blood lipids and preventing thrombosis^35,36^. The content of C18:3 in the mutant was slightly lower than that in the wild type. Since C18:3 has an unpleasant smell and tastes slightly worse^37^, a slightly lower content of C18:3 in leaves tastes better when consumed as a vegetable (Supplementary Fig. 2). The second most abundant composition was C16:0 (Table 1), a kind of saturated fatty acid and mainly used for food processing, cosmetics, cleaning products, biofuels, and animal feed^38^. In fresh or dry vegetables, a certain amount of C16:0 makes it uneasy to be oxidized and thus has a longer fresh-keeping period or shelf life. So, in this study, the mutant leaves with 15.652 mg/g C16:0 were more suitable for fresh cooking, while the wild-type leaves (20.014 mg/g) were more suitable for dry or caned usage. The contents of C11:0 and linoleic acid C18:2 ranged from 4.0 to 4.9 mg/g, and the other ten fatty acid compositions were below 1.6 mg/g (Table 1). Taken together, the mutant JQA leaf has a high proportion of unsaturated fatty acids beneficial to human health.

The sum of unsaturated fatty acids in the mutant leaves was 32.0 mg/g (accounting for 56.4% of total fatty acids), which was higher than that of saturated fatty acids (24.8 mg/g, 43.6% of total). In the wild-type JQB, these values were respective 39.5 mg/g (56.4% of total) and 30.0 mg/g (43.6% of total) (Table 1 and Supplementary Fig. 3). The most abundant unsaturated fatty acids were C18:3, C18:2, and C18:1 in the wild type leaves, accounting for 47.3% of the total amount of the fourteen fatty acids, and the saturated fatty acids were C16:0, C11:0, and C18:0 (accounting for 38.1% of the total). The contents of the above fatty acids in the mutant JQA leaves were lower but not statistically significant. This indicated that the proportion of unsaturated fatty acids and saturated fatty acids in the leaves was very stable both in the mutant and the wild type. This is good for the preservation and non-perishability of the leaves. Our results indicate that sesame leaf is also a good source of oil.

### Soluble protein and amino acids

The measured results showed that the content of soluble protein in the leaves of JQA and JQB was 0.41 mg/g and 0.72 mg/g, respectively (Table 2), which is lower than that of color pepper (10.3 mg/g) and other vegetables^39^. The total contents of seventeen amino acids detected in the leaves of JQA and JQB were 18.64 and 23.45 mg/g, respectively, being lower in the mutant JQA than in wild-type JQB. The leaves of JQA had lower values for each amino acid compared to JQB. The total contents of eight essential amino acids (namely Thr, Try, Val, Met, Phe, Ile, Leu, and Lys) accounted for 43.3% and 44.1% of the sum of 17 amino acids in JQA and JQB, respectively, and no significant difference was found between the two materials (Table 2).

**Table 2:** Soluble protein and amino acids in sesame leaves (mg/g).

Parameters^a^	Wild type JQB	Mutant JQA^b^
Soluble protein	0.72 ± 0.09	0.41 ± 0.04**
**Hydrolytic amino acid**		
Asp	2.54 ± 0.13	2.37 ± 0.22
Glu	3.05 ± 0.32	2.48 ± 0.33
Ser	0.94 ± 0.21	0.72 ± 0.10
His	0.56 ± 0.12	0.43 ± 0.07
Gly	1.45 ± 0.12	1.08 ± 0.24
Thr	1.04 ± 0.09	0.84 ± 0.26
Arg	1.35 ± 0.20	1.09 ± 0.21
Ala	1.46 ± 0.15	1.12 ± 0.20
Try	0.73 ± 0.16	0.62 ± 0.26
Cys	0.06 ± 0.02	0.02 ± 0.01*
Val	1.62 ± 0.28	1.32 ± 0.20
Met	0.16 ± 0.05	0.13 ± 0.04
Phe	1.51 ± 0.23	1.18 ± 0.12
Ile	1.38 ± 0.38	1.06 ± 0.16
Leu	2.33 ± 0.38	1.70 ± 0.13
Lys	1.57 ± 0.26	1.19 ± 0.22
Pro	1.65 ± 0.18	1.23 ± 0.32
Total	23.45 ± 3.08	18.64 ± 2.92
Subtotal of E	10.36 ± 1.64	8.08 ± 1.326
Subtotal of N	13.08 ± 1.46	10.56 ± 1.62
Subtotal of C	1.91 ± 0.33	1.53 ± 0.28
E (%)	44.11 ± 1.39	43.31 ± 1.10
N (%)	55.89 ± 1.47	56.68 ± 1.12
C (%)	8.13 ± 0.42	8.18 ± 0.25
E/N ratio	0.78 ± 0.05	0.76 ± 0.03

^a^*Asp* aspartic acid, *Glu* glutamine, *Ser* serine, *His* histidine, *Gly* glycine, *Thr* threonine, *Arg* arginine, *Ala* alanine, *Try* tyrosine, *Cys* cysteine, *Val* valine, *Met* methionine, *Phe* phenylalanine, *Ile* isoleucine, *Leu* leucine, *Lys* lysine, *Pro* proline, *E* essential amino acids, *N* non-essential amino acids, *C* essential amino acids only for children.

^b^Values are means ± standard deviation (*n* = 3). Data in the same row followed by * and ** are significantly (*P* < 0.05) and very significant (*P* < 0.01) different, respectively.

The content of all amino acids, essential amino acids, non-essential amino acids, and essential amino acids only for children (namely Arg and His) was noted as T, E, N, and C, respectively. The mass fraction of essential amino acids to total amino acids (E/T), the quality score of essential amino acids only for children to total amino acids (C/T), and the mass ratio of essential amino acids to non-essential amino acids (E/N) were then calculated. As can be seen from Table 2, the E values varied from 43.3% to 44.1%, and the E/N from 0.76 to 0.78, which were similar between the wild type and the mutant.

According to modern nutritional theory, the nutritional value of a protein is closely related to the amino acid composition of the protein^40^. The more the amino acid composition of a food protein enters into the human body, the higher the nutritional value upon digestion and absorption^39^. As proposed by FAO/WHO in 1973, a high-quality protein should have an E/T value of about 40% and an E/N value of over 0.60 (https://www.fao.org/nutrition/en/). In this study, the E/T values of the wild type and mutant were all exceeding 40%, and the E/N values were all exceeding 0.60 (Table 2); therefore, both mutant JQA and the wild type JQB meet the ideal protein requirements and are suitable for consumption in terms of amino acids.

### Macro- and micro-elements

The macro-elements and micro-elements in the leaves of the mutant and the wild type were determined. It is evident from Table 3 that nitrogen (N), calcium (Ca), potassium (K), and magnesium (Mg) were the main constituents of sesame leaves. N and Ca were significantly present in leaves, with contents varying from 30.9 to 31.9 mg/g and 18.5 to 21.6 mg/g, respectively. K and Mg represented the third and fourth major elements (> 2.2 mg/g). The Ca content in mutant leaves (18.5 mg/g) was especially higher in comparison to K (16.1 mg/g) and Na (0.08 mg/g), and the value of K/Na was 142 and 207 in the wild type, and the mutant, respectively, which is positive from a nutritional perspective. The phosphorus (P) level obtained in the present study was very low, but the content of K in the mutant leaves was significantly higher than in the wild type (11.7 mg/g). K functions in regulating the appropriate osmotic pressure in cells and the acid/base balance of body fluids^41^. It is also involved in the metabolism of sugars and proteins in cells and thus is important for physiological functioning^41^. Compared with other vegetables such as white radish, cabbage, spinach, lettuce, and rapeseed, the contents of Ca and Selenium (Se) in sesame leaves were high, especially Se, suggesting that sesame leaves offer a rich source of these minerals (Supplementary Table 1). In all, the above data exhibited that the mutant JQA leaf is rich in both macro and micro elements required for health.

**Table 3:** The contents of macro- and micro-elements in sesame leaves.

Elements^a^	Wild type JQB	Mutant JQA^b^
**Macro-elements (mg/g)**		
N	30.85 ± 2.09	31.89 ± 1.55
P	0.30 ± 0.02	0.37 ± 0.04
K	11.74 ± 0.85	16.13 ± 0.88**
Na	0.08 ± 0.01	0.07 ± 0.01
Ca	21.57 ± 1.02	18.52 ± 0.75*
Mg	3.05 ± 0.10	2.18 ± 0.16**
**Micro-element (μg/g)**		
Mn	58.13 ± 7.25	37.13 ± 3.55*
B	25.10 ± 1.10	19.03 ± 2.05
Cu	16.31 ± 0.98	12.80 ± 0.98*
Zn	19.62 ± 2.04	18.15 ± 1.05
Se	0.41 ± 0.06	0.32 ± 0.02

^a^*N* nitrogen, *P* phosphorus, *K* potassium, *Na* sodium, *Ca* calcium, *Mg* magnesium, *Mn* manganese, *B* boron, *Cu* copper, *Zn* zinc, *Se* selenium.

^b^Values are means ± standard deviation (*n* = 3). Data in the same row followed by * and ** are significantly (*P* < 0.05) and very significant (*P* < 0.01) different, respectively.

### Total sugar and vitamins

The results showed that the content of total sugar (30.21–32.46 mg/g), reducing sugar (12.53–13.42 mg/g), and soluble sugar (17.36–18.82 mg/g) were abundant and did not differ significantly between the wild type and the mutant (Table 4). Moreover, the sesame leaves were also rich in vitamin B_6_ (24.46–38.54 mg/g) and B_2_ (11.85–14.40 mg/g). Compared to the wild type, the contents of vitamin C, vitamin D, and vitamin B_2_ in the mutant JQA were significantly or very significantly higher, while the content of vitamin B_6_ was markedly lower (*P* < 0.01). The contents of other vitamins (A and B_1_) were similar between JQA and JQB. All of the vitamins are vital to human health^42,43^. For instance, vitamin A is found in fish liver oil, animal liver, and green vegetables; the lack of vitamin A can result in night blindness^44^. Vitamin B_2_ is found in yeast, vegetables, and eggs, and a lack of it can cause oral inflammation (oral ulcer)^45^. Vitamin C protects against scurvy and it is an important vitamin widely found in fresh fruits and vegetables; as a highly active substance, it participates in many metabolic processes^46^. Vitamin D deficiency can lead to rickets in children and osteopathy in adults^47^. Thus, our data suggested that the leaves of the JQA mutant are a good source of vitamins, especially for those people with deficiencies in vitamins A, C, D, and B_2_.

**Table 4:** Total sugar, reducing sugar, total soluble sugar, and vitamins contents in sesame leaves (mg/g).

Materials	Wild type JQB	Mutant JQA
Total sugar	32.46 ± 1.94	30.21 ± 0.85
Reducing sugar	13.42 ± 0.71	12.53 ± 1.43
Soluble sugar	18.82 ± 1.49	17.36 ± 1.33
**Vitamin**		
C	1.24 ± 0.08	1.69 ± 0.13*
A	0.80 ± 0.04	0.97 ± 0.10
D	0.81 ± 0.03	1.33 ± 0.08**
B_1_	1.86 ± 0.12	1.54 ± 0.21
B_2_	11.85 ± 0.99	14.40 ± 1.46*
B_6_	38.54 ± 1.55	24.46 ± 1.78**

Values are means ± standard deviation (*n* = 3). Data in the same row followed by * and ** are significantly (*P* < 0.05) and very significant (*P* < 0.01) different, respectively.

### Biological traits and other nutritional indicators

We measured the biological traits of the wild-type and mutant (Table 5), and the results indicated that leaf length (17.62–17.92 cm) was similar between these two lines, but the width of the mutant leaf (19.56 cm) was much bigger than that of the normal leaf (16.12 cm). Additionally, the fresh weight per leaf in the mutant (15.61 g) was greater than in the wild type (12.21 g). Thus, the fresh leaves yield of the mutant was > 20% higher than that of the wild type (Table 5). The water content was slightly higher in the mutant (86.5%) than in the wild type, whereas the crude fiber (0.36%), cellulose (21.37%), and lignin (0.55%) contents were slightly lower than in the wild type. The mutant leaves tasted tender when cooked, which was expected because they were more tender than those of the wild type by visual inspection in the field, with the latter having a rough leaf appearance. The total flavone content in the mutant (65.67 mg/g) was slightly higher than in the wild type. Flavone has a strong antioxidant capacity and radical scavenging ability, and thus it is good for health^48^. In summary, the leaves of the mutant are more suitable for cooking as a vegetable because of their nutrition and palatability (Supplementary Fig. 2).

**Table 5:** The biological and nutritional characteristics of sesame leaves.

Traits	Wild type JQB	Mutant JQA
Leaf length (cm)	17.66 ± 1.40	17.65 ± 1.69
Leaf width (cm)	16.17 ± 0.95	19.59 ± 1.20*
Water content (%)	85.14 ± 2.59	86.52 ± 1.96
Fresh leaf yield (t/ha)	10.51 ± 1.13	13.32 ± 1.45*
Crude fiber (%)	0.38 ± 0.04	0.36 ± 0.02
Cellulose (mg/g)	23.62 ± 0.71	21.378 ± 1.66
Hemicellulose (mg/g)	24.15 ± 1.09	26.09 ± 1.03
Lignin (%)	0.77 ± 0.11	0.56 ± 0.09
Total flavone (mg/g)	63.18 ± 4.13	66.71 ± 2.83

Values are means ± standard deviation (*n* = 3). Data in the same row followed by * and ** are significantly (*P* < 0.05) and very significant (*P* < 0.01) different, respectively.

Vegetables are not only rich in vitamins, minerals, dietary fiber, and natural antioxidants, but are also an essential and non-negligible source of dietary protein. The protein content of vegetables is usually between 1 and 4%, and therefore they are considered to be low-protein products. However, the daily intake of vegetables in diets is relatively high, and the protein provided to the human body is about 8–10% of the actual dietary requirements^49^. In recent years, the exploitation and utilization of plant protein resources have gained increased attention. However, few studies have reported on the nutritional value of vegetable proteins. Our results show that sesame wrinkled leaves are a potentially good source of dietary protein. The nutrient composition of the JQA mutant sesame leaves was superior to that of cultivated vegetables such as radish, Chinese cabbage, rapeseed, cauliflower, spinach, and lettuce (Supplementary Table 1). Our research confirmed that the protein and nutritional value of sesame mutant JQA leaves are higher than that of ordinary vegetables. The amino acid content of sesame JQA leaves ranged from 18.56 to 23.37 mg/g, and the content of essential amino acids was higher than 43%. Due to the balanced amino acid, sesame JQA leaves tasted fresher and had a delicate texture.

In this study, the content of micronutrients in the sesame JQA leaves was exceptionally high. The contents of mineral elements detected in sesame leave ranked in descending order as N > Ca > K > Mg > P > Na > Mn > B > Zn > Cu > Se (Table 3). Micronutrients are essential nutrients that play an important role in the physiological functioning of the human body. Compared with common leafy vegetables, the contents of Ca, Se, crude protein, and crude fiber were higher in the sesame leaves. Ca enhances the process of inhibition on the surface of the brain, regulates the imbalance between excitement and inhibition, reduces inflammation, reduces swelling, resist allergy and detoxification, and is negatively correlated with hypertension^50^. Se is an essential trace element in the human body and can enhance antioxidant capacity, boost immunity, and prevent viral diseases. Crude fiber can help with digestion and eliminate waste in the body, to reduce harmful toxins in the body^51^. The sesame leaves contained a high amount of K, therefore representing a high-K and low-Na food. Consumption of sesame leaves would help bring down blood pressure, reduce cardiovascular diseases, and promote sugar metabolism.

The content of crude fiber in the leaves of the mutant was lower than that of the wild type, because the mutant leaves appeared younger, more tender, and thicker, rendering them easier to chew and more suitable for consumption. Vitamin C has the reputation of being a “universal vitamin”, which can enhance the immune function of the human body, prevent and treat anemia, and maintain the normal functioning of bones and teeth^52^. In this study, the content of vitamin C in the JQA mutant was 35.17% higher than that in the wild type. Sesame leaves are therefore ideal for supplementing vitamin C in the diet.

## Conclusions

Sesame is traditionally an oil crop that can grow well in environmentally stressed conditions with huge biomass, which promotes the idea that sesame leaves are a potential source of easily-accessible green vegetables for the poor population. Here we systematically evaluated the nutritional value of sesame leaves, and the results strongly support that JQA mutant is a new source of edible leafy vegetables. The nutrient composition of the sesame mutant leaves was superior to that of other cultivated vegetables. The leaves of JQA are a good source of vitamins, especially vitamins A, C, D, and B_2,_ and minerals as well (i.e. K, Ca, and Se). Moreover, it also exhibited balanced hydrolytic amino acids, and high levels of crude lipid, saturated acid, unsaturated acid, and lignin, implying that it is a healthy and beneficial vegetable for human consumption, especially for the low-income population.

## Supplementary Information

Supplementary Information.

## Data Availability

All data generated or analysed during this study are included in this published article and its supplementary information files, which are available from the corresponding author on reasonable request.
