# Supplementary material for: A novel wrinkled-leaf sesame mutant as a potential edible leafy vegetable rich in nutrients
Source: Sci Rep. 2022 Nov 2;12:18478. doi: 10.1038/s41598-022-23263-0 (PMC9630307; doi:10.1038/s41598-022-23263-0)
Supplement: Supplementary file 1 — Supplementary Information. [file 41598_2022_23263_MOESM1_ESM.docx]

**Supplementary data for Liu et al.**

**A novel wrinkled-leaf sesame mutant as a potential edible leafy vegetable rich in nutrientss**

Hongyan Liu^1^, Fang Zhou^1^, Ting Zhou^1^, Yuanxiao Yang^1^, Yingzhong Zhao^1,^*

^1^Key Laboratory of Biology and Genetic Improvement of Oil Crops, Ministry of Agriculture, Oil Crops Research Institute of Chinese Academy of Agricultural Sciences, Wuhan, Hubei 430062, China

**E-mail:**

H.Y. Liu liuhongyan@caas.cn;

Y.Z. Zhao zhaoyz63@163.com

* Corresponding author:

Y.Z. Zhao, Email: zhaoyz63@163.com; Tel: 86-027-86833625

**Supplementary Table 1 Comparison of amount and component of nutrition elements in sesame leaves and other vegetables**

| Vegetables | Calcium  (mg/g) | Selenium  (mg/kg) | Crude protein  (%) | Crude fiber  (%) | Fructose  (mg/g) | Reference ^b^ |
| --- | --- | --- | --- | --- | --- | --- |
| Sesame ^a^ | 20.1 | 0.365 | 3.14 | 0.37 | 12.94 | This study |
| White Radish | 3.00 | 0.003 | 0.44 | 0.69 | 4.03 | OCRI |
| Chinese cabbage | 1.87 | 0.003 | 0.71 | 0.47 | 2.51 | OCRI |
| Cauliflower | 2.47 | 0.006 | 1.25 | 0.78 | 5.03 | OCRI |
| Red bolt | 4.34 | 0.008 | 3.56 | 1.78 | 8.91 | OCRI |
| Spinach | 5.57 | 0.009 | 2.02 | 0.74 | 5.30 | OCRI |
| Lettuce | 5.81 | 0.004 | 1.38 | 1.29 | 6.14 | OCRI |
| Rapeseed shoot (Zhongyougaoxi 1) | 16.49 | 0.020 | 2.38 | 1.33 | 15.12 | OCRI |

^a^ the mean of JQA and JQB. Crude protein was inferred from N% (converted from mg/g).

^b^ OCRI report (http://www.ocri.caas.cn).


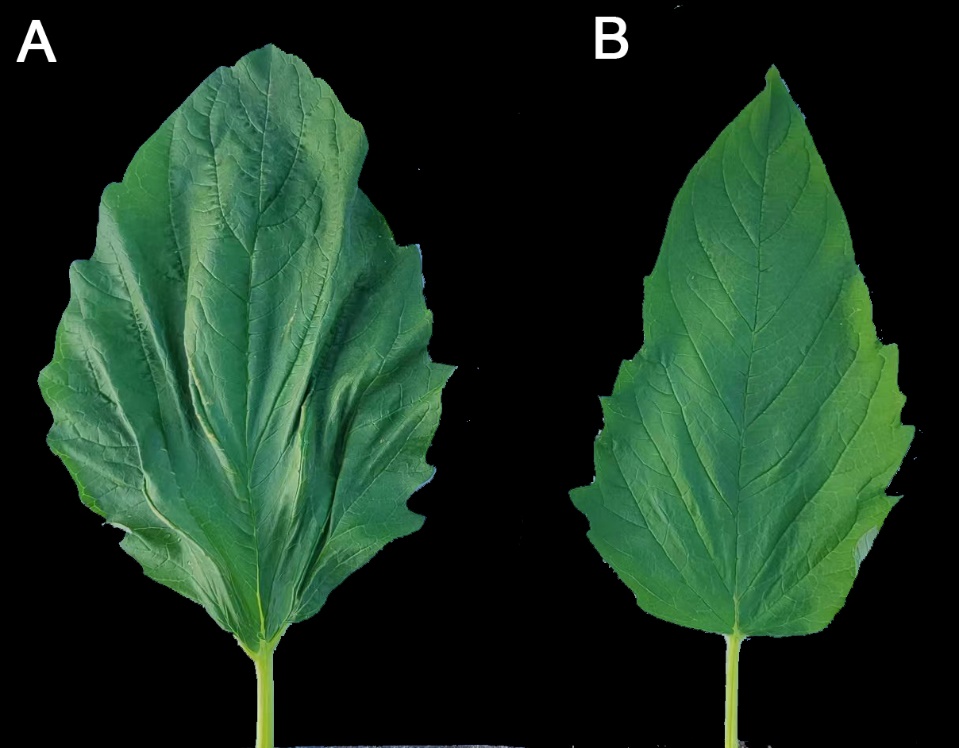


**Supplementary Figure 1 Morphology of fresh sesame leaves.** A, wrinkled leaf of JQA; B, normal leaf of JQB.


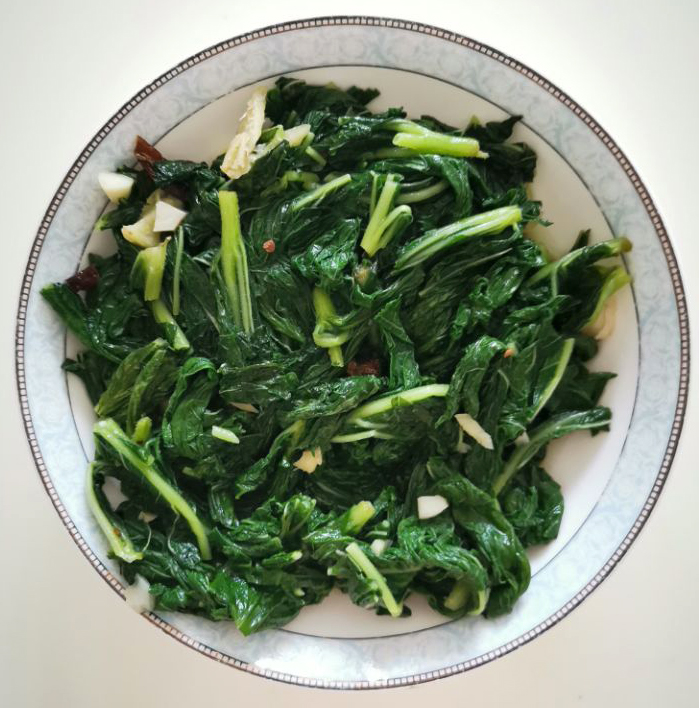


**Supplementary Figure 2 A cold dish of sesame leaves (green) with some garlic (white) and pepper (red).**


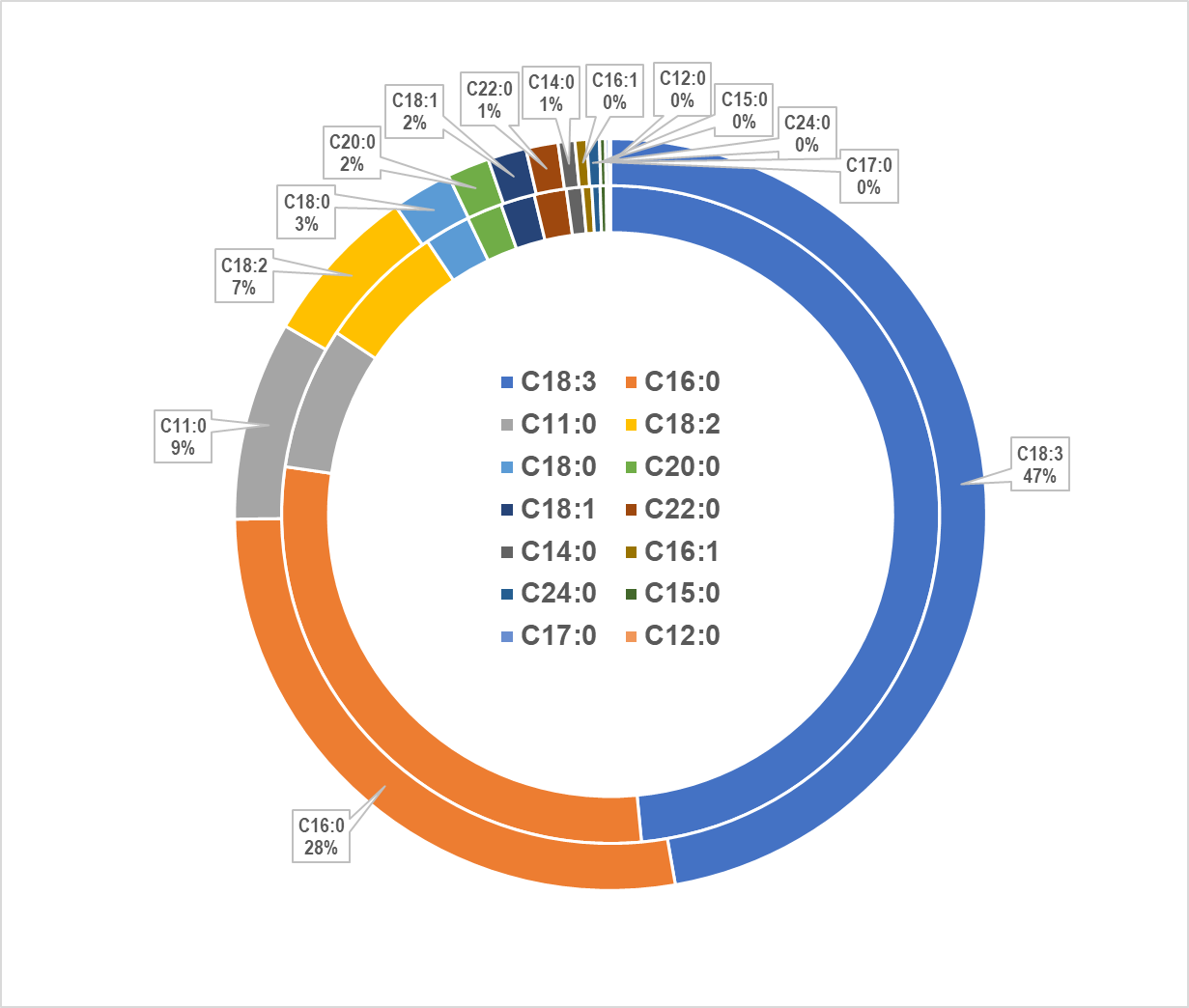


**Supplementary Figure 3 Proportion (%) of 14 fatty acid compositions detected in normal sesame leaves JQB (outer circle) and wrinkled leaves JQA (inner circle).**
